# One Health genomic insights into environmental and animal reservoirs of community-associated *Clostridioides difficile* in Japan

**DOI:** 10.1128/aem.00397-26

**Published:** 2026-05-11

**Authors:** Sadako Yoshizawa, Kohji Komori, Kotaro Aoki, Tomoka Sawa, Yoshimasa Sasaki, Yasuhisa Kurosaki, Yoshiteru Murata, Tomoko Kasai, Nobuaki Mori, Tetsuo Asai, Yoshikazu Ishii, Kazuhiro Tateda

**Affiliations:** 1 Department of Laboratory Medicine, Toho University School of Medicine, Tokyo, Japan; 2 Department of Microbiology and Infectious Diseases, Toho University School of Medicine, Tokyo, Japan; 3 Division of Collaborative Regional Infection Control, Department of Community Well-being, Toho University School of Medicine, Tokyo, Japan; 4 Department of Paediatrics, Toho University School of Medicine, Tokyo, Japan; 5 Division of Veterinary Science, Department of Veterinary Medicine, Obihiro University of Agriculture and Veterinary Medicine, Hokkaido, Japan; 6 Kurosan Animal Clinic, Kagoshima, Japan; 7 Research and Education Center for Prevention of Global Infectious Diseases of Animals, Tokyo University of Agriculture and Technology, Tokyo, Japan; 8 Murata Animal Hospital, Chiba, Japan; 9 Muni Veterinary Hospital, Tokyo, Japan; 10 Department of Infectious Diseases, Showa University School of Medicine, Tokyo, Japan; 11 United Graduate School of Veterinary Medicine, Gifu University 12785, Gifu, Japan; 12 Center for the Planetary Health and Innovation Science (PHIS), The IDEC Institute, Hiroshima University, Hiroshima, Japan; Centers for Disease Control and Prevention, Atlanta, Georgia, USA

**Keywords:** *Clostridioides difficile*, outpatients, soil, livestock, companion animals, wild animals, Japan, molecular epidemiology, one health

## Abstract

*Clostridioides difficile* is a well-recognized cause of antibiotic-associated colitis. The highly pathogenic PCR ribotype(RT)027/sequence type(ST)1 strain has been prevalent since 2002, particularly in Europe and the United States, whereas strains detected in Japan primarily comprise RT018/ST17, RT369/ST81, and RT002/ST8. Community-associated *C. difficile* infection (CA-CDI) has increased in recent years, and an association with livestock, food, and companion animals has been suggested. This study investigated the molecular epidemiology of *C. difficile* in Japan, focusing on isolates from diarrheal outpatients in comparison with animal and environmental sources. A total of 401 samples were collected, including park soil and fecal samples from livestock, companion animals, wild animals, and outpatients. Overall, 63 strains were isolated. High isolation rates were observed in soil samples (19/37, 51%) and companion animals (19/64, 30%), of which 68% and 37% were toxigenic, respectively. Whole-genome sequencing revealed ST2 as the most common sequence type, followed by ST42 and ST15. No single sequence type predominated among isolates from diarrheal outpatients, and six patients met the epidemiological definition of CA-CDI. Core-genome single-nucleotide polymorphism (SNP) analysis identified close genetic relationships in two pairs of ST42 strains—one between a patient-derived isolate and park soil, and another between a canine-derived isolate and soil from a different park—using a hypothetical transmission-exclusion cutoff of <13 SNPs. These findings suggest the presence of shared environmental reservoirs linking humans, animals, and soil, rather than indicating the direction of transmission. The study highlights the importance of considering environmental reservoirs within a One Health framework for CA-CDI in Japan.

**IMPORTANCE:**

*Clostridioides difficile* infection (CDI) is a widely recognized cause of antibiotic-associated diarrhea, and reports of community-associated CDI (CA-CDI) have risen in recent years. Understanding how *C. difficile* circulates outside healthcare settings is, therefore, an important public health challenge. In this study, we analyzed *C. difficile* isolates from outpatients with diarrhea, companion animals, and environmental sources, including soil from public parks. The organism was frequently detected in soil and pets, and genomic analyses identified closely related strains across human, animal, and soil samples. Although these findings do not indicate transmission direction, they suggest that environmental reservoirs may contribute to the broader ecology of *C. difficile* in the community. Our results underscore the importance of a One Health perspective integrating human, animal, and environment together when studying CA-CDI and highlight the need for further research to better understand how *C. difficile* persists and spreads outside hospital settings.

## INTRODUCTION

*Clostridioides difficile* (*C. difficile*) was first isolated from the feces of a healthy newborn in 1935 and has been recognized as the cause of antibiotic-associated enteritis since 1978 (1, 2). In the early 2000s, a hypervirulent strain—RT027/ST1 (also known as the BI/027/NAP1 strain)—emerged in North America and Europe, characterized by increased toxin A and B production, binary toxin (CDT) production, enhanced sporulation, *tcdC* mutations, and fluoroquinolone resistance tendency (3–6). Another virulent lineage, RT078/ST11, has also been linked to severe outbreaks (7). In contrast, the molecular epidemiology of *C. difficile* in Japan differs significantly. RT027/ST1 and RT078/ST11 are rarely detected, whereas strains such as RT018/ST17, RT369/ST81, and RT002/ST8 are more prevalent (8–11). The incidence of *C. difficile* infection (CDI) in Japan is estimated at 7.4 per 10,000 patient-days, with asymptomatic colonization in approximately 7.6% of healthy individuals (12).

While CDI has traditionally been linked to healthcare settings and antibiotic use, reports of community-associated CDI (CA-CDI) have increased worldwide (13–16). In the United States, CA-CDI accounts for 41% of all CDI cases (17). In Australia, recent national surveillance data indicate that the proportion of CA-CDI has increased substantially, reaching approximately 80% in recent years (18), while Europe has reported lower but increasing proportions (32.7%) (19). A multicenter prospective study in Japan reported that 9.1% of CDI cases were community-associated (20). CA-CDI often occurs in younger, otherwise healthy individuals with no recent antibiotic use or healthcare exposure (21, 22).

Growing evidence suggests environmental reservoirs, livestock, companion animals, and food as contributing factors to CA-CDI transmission, with whole-genome sequencing (WGS) analyses demonstrating genetic relatedness among strains from different sources (16, 23–30). In Japan, *C. difficile* has been detected in pigs and piglets (0.8%–57.5%), calves (17%), and canines (30%), but not in cattle; data regarding felines remain lacking (31–35). In one study, 47.5% of sandbox samples from Ishikawa Prefecture in Japan tested positive for *C. difficile* (36).

Despite these findings, the role of animals and the environment in CA-CDI transmission remains poorly understood in Japan. To address this gap, we examined the prevalence of *C. difficile* among companion animals, livestock, wild animals, and park soil. Furthermore, we conducted whole-genome single-nucleotide polymorphism (SNP) analysis to assess the genetic relatedness between environmental/animal isolates and those obtained from diarrheal outpatients, aiming to identify potential transmission pathways of CA-CDI.

## RESULTS

### Isolation of *C. difficile* and toxin gene possession status

**Fig 1:**
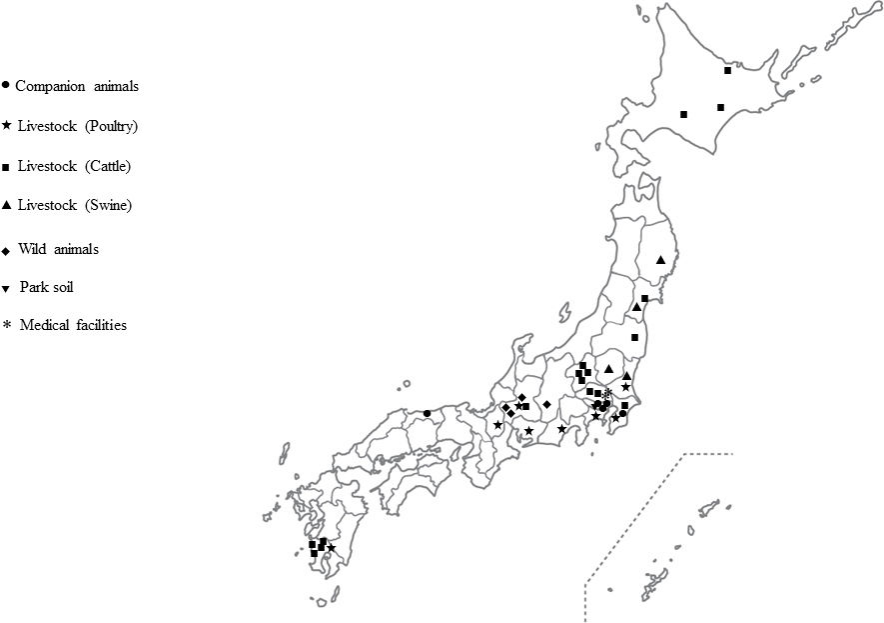
Geographic distribution of sample collection sites in Japan. Sampling locations for companion animals, livestock (cattle, poultry, and swine), wild animals, park soil, and medical facilities are shown. Symbols indicate the approximate locations of sample collection sites for each category, as described in the legend.

A total of 401 samples were collected in Japan from companion animals, livestock (cattle, poultry, and swine), park soil, wild animals, and outpatients (Table 1; Fig. 1). During the study period, *C. difficile* was isolated from 12 diarrheal outpatient samples, of which six isolates were toxin gene positive and diagnosed as CA-CDI (Table S1, No. 29, No. 35, No. 39, No. 41, No. 56, No. 59). Fecal samples from 34 canines and 30 felines were analyzed. *C. difficile* was isolated from 47% of canine samples, with a median age of 4.5 years (IQR: 0.8–6). Among the canine isolates, 37.5% were toxin gene-positive. In felines, the isolation rate was 10%, with a median age of 8 years (range: 0.2–16 years). A toxin gene-positive strain was detected in a 2-month-old kitten.

**TABLE 1:** Prevalence of *Clostridioides difficile* across sample sources in Japan^*a*^

Source category	Type of sample	No. of samples	Overall prevalence (%)	Toxigenic prevalence (%)	Sampling location
Companion animals	Felines	30	3 (10)	1 (33.3)	3 veterinary clinics (2 in Kanto, 1 in Kansai)
Companion animals	Canines	34	16 (47)	6 (37.5)	3 veterinary clinics (2 in Kanto, 1 in Kansai), 2 households in Tokyo
Livestock	Cattle	135	15 (11.1)	7 (46.7)	17 facilities (3 in Hokkaido, 2 in Tohoku, 1 in Gifu, 7 in Kanto, 4 in Kagoshima)
Livestock	Poultry	56	0	0	8 facilities (3 in Kanto, 1 in Kagoshima, 1 in Shiga, 1 in Gifu, 1 in Aichi, 1 in Shizuoka)
Livestock	Swine	40	0	0	4 facilities (2 in Kanto, 2 in Tohoku)
Environment	Soil	37	19 (51.4)	13 (68)	8 parks (Tokyo metropolitan area)
Wild animals	Mixed wildlife	51	4 (7.8)	0	Multiple environments in Gifu (forest edges, agricultural fields, riverbeds, and peri-urban green spaces) and a village site in Nagano
Humans	Patients	12	12	6	2 facilities in Tokyo metropolitan area
	Total	401	63 (15.7)	40 (63.5)	

^*a*^ Overall prevalence (%) indicates the proportion of samples positive for *C. difficile*. Toxigenic prevalence (%) indicates the proportion of isolates carrying toxin genes among detected strains. Sampling locations are specified to highlight geographic diversity. Kanto region includes Tokyo, Kanagawa, Chiba, Saitama, Ibaraki, Tochigi, and Gunma prefectures. Kansai region includes Osaka, Kyoto, Hyogo, Nara, Shiga, and Wakayama prefectures. Tohoku region includes Aomori, Iwate, Miyagi, Akita, Yamagata, and Fukushima prefectures. Mixed wildlife represents raccoon dogs, foxes, martens, deer, cats, civets, badgers, raccoons, and nutria.

Samples from livestock included 135 cattle, 56 poultry, and 40 swine. The cattle had a median age of 14.3 months (IQR:2.5–27.6), with an isolation rate of 11.1% and toxin-gene positivity of 77.8%. *C. difficile* was not isolated from poultry or swine; the median ages were 19.2 months (IQR: 18.3–21.5) for poultry and 4.5 months (IQR: 3–6) for swine. Fecal samples from 51 wild animals were obtained, including raccoon dogs, foxes, martens, deer, stray cats, civets, badgers, raccoons, and nutria. Four non-toxigenic *C. difficile* strains were isolated from a nutria, raccoon dog, marten, and badger, respectively. Additionally, 37 soil samples were collected from 8 urban parks, with a *C. difficile* detection rate of 51.4%, of which 68% were positive for toxin genes (Table 1; Table S1).

### STs and toxin-encoding gene profiles

A total of 63 isolates were examined using multilocus sequence typing (MLST) analysis (Table 2). Among these, ST2 was the most prevalent, accounting for 11 isolates, of which 7 originated from soil samples. ST42 was the second most common type, with 9 isolates, including 6 from soil. ST15 accounted for 6 isolates, 4 of which were obtained from canine sources. In addition, ST100, ST11, and ST203 were each detected in multiple isolates, whereas the remaining sequence types were identified sporadically.

**TABLE 2:** Multilocus sequence typing and toxin-encoding gene profiles of *C. difficile* strains

Sequence type (ST)	Estimated RT^*a*^	No. of strains						Total no. of strains	Toxin gene profile
		Patients	Felines	Canines	Cattle	Soil	WA^*b*^		
ST2	RT014/020/076/220		1	2	1	7		11	*tcdA ^+^ tcdB ^+^ cdtA^−^ cdtB^−^*
ST42	RT106/118/174	1		2		6		9	*tcdA ^+^ tcdB ^+^ cdtA^−^ cdtB^−^*
ST15	RT010			4		2		6	Nontoxigenic
ST100	NA^*c*^	2		1		2	1	6	Nontoxigenic
ST203	NA	2	1	1		1		5	Nontoxigenic
ST11	RT078/126/127/033				5			5	*tcdA ^+^ tcdB ^+^ cdtA ^+^ cdtB* ^+^
ST26	RT039/140			3				3	Nontoxigenic
ST28	NA	1		1				2	Nontoxigenic
ST3	RT001/009/072/220			1				1	Nontoxigenic
ST37	RT017	1						1	*tcdA ^+^ tcdB ^+^ cdtA^−^ cdtB^−^*
ST47	NA	1						1	*tcdA ^+^ tcdB ^+^ cdtA ^+^ cdtB* ^+^
ST58	NA				1			1	*tcdA ^+^ tcdB ^+^ cdtA^−^ cdtB^−^*
ST81	NA	1						1	*tcdA ^+^ tcdB ^+^ cdtA^−^ cdtB^−^*
ST101	NA				1			1	Nontoxigenic
ST109	NA						1	1	Nontoxigenic
ST185	NA			1				1	*tcdA ^+^ tcdB ^+^ cdtA^−^ cdtB^−^*
ST224	NA	1						1	*tcdA ^+^ tcdB ^+^ cdtA^−^ cdtB^−^*
ST297	NA				1			1	Nontoxigenic
ST918	NA	1						1	Nontoxigenic
ST919	NA	1						1	*tcdA^−^ tcdB ^+^ cdtA^−^ cdtB^−^*
ST920	NA					1		1	Nontoxigenic
ST921	NA						1	1	Nontoxigenic
ST922	NA						1	1	Nontoxigenic
ST1289	NA		1					1	Nontoxigenic
Total		12	3	16	9	19	4	63	

^*a*^ Estimated RT was determined by references 12 and 37.

^*b*^ WA, wild animal.

^*c*^ NA, not assigned, as information on the relationship between ST and ribotype (RT) was not available.

Among the toxigenic profiles, ST2, ST42, ST11, ST37, ST47, ST58, ST81, ST185, and ST224 were positive for *tcdA* and *tcdB* (*tcdA^+^tcdB^+^*). ST 919 was positive for *tcdB* only (*tcdA^−^ tcdB^+^*). Additionally, ST11 and ST47 were positive for *cdtA* and *cdtB* (*cdtA^+^cdtB^+^*). Isolates from diarrheal outpatients and felines showed relatively diverse ST distributions. In contrast, four ST15 isolates and three ST26 isolates originated from canines, while five ST11 isolates were recovered from cattle. Notably, ST2 and ST42 were frequently detected in soil, with 7 and 6 isolates, respectively.

### Antimicrobial resistance determinants of *C. difficile* isolates

Amino acid substitutions associated with fluoroquinolone resistance were identified in several strains. Five strains carried a threonine-to-isoleucine substitution at position 82 of GyrA (GyrA Thr82Ile), with each strain belonging to a different ST. Additionally, substitutions at position 139 of GyrB (GyrB Ile139Arg/Val) were observed in 12 strains: 11 strains carrying the Ile139Arg mutation were all ST2, whereas one strain with the Ile139Val mutation was classified as ST297. At position 366 of GyrB (GyrB Ser366Arg/Val), substitutions were identified in 14 strains, including 5 with a Ser366Val substitution, all of which were classified as ST11. Additionally, 2 strains displayed a substitution at position 426 of GyrB (GyrB Asp426Val), each belonging to different STs. RpoB and RpoC, which are associated with fidaxomicin resistance, did not have the mutations previously reported to contribute to resistance (RpoB V1143, Q1149; RpoC R89, R326). Moreover, three strains were found to carry the *erm*(B) gene, and one strain harbored *erm*(T) (Table S1).

### Investigation of strain relatedness using core-genome SNP analysis

**Fig 2:**
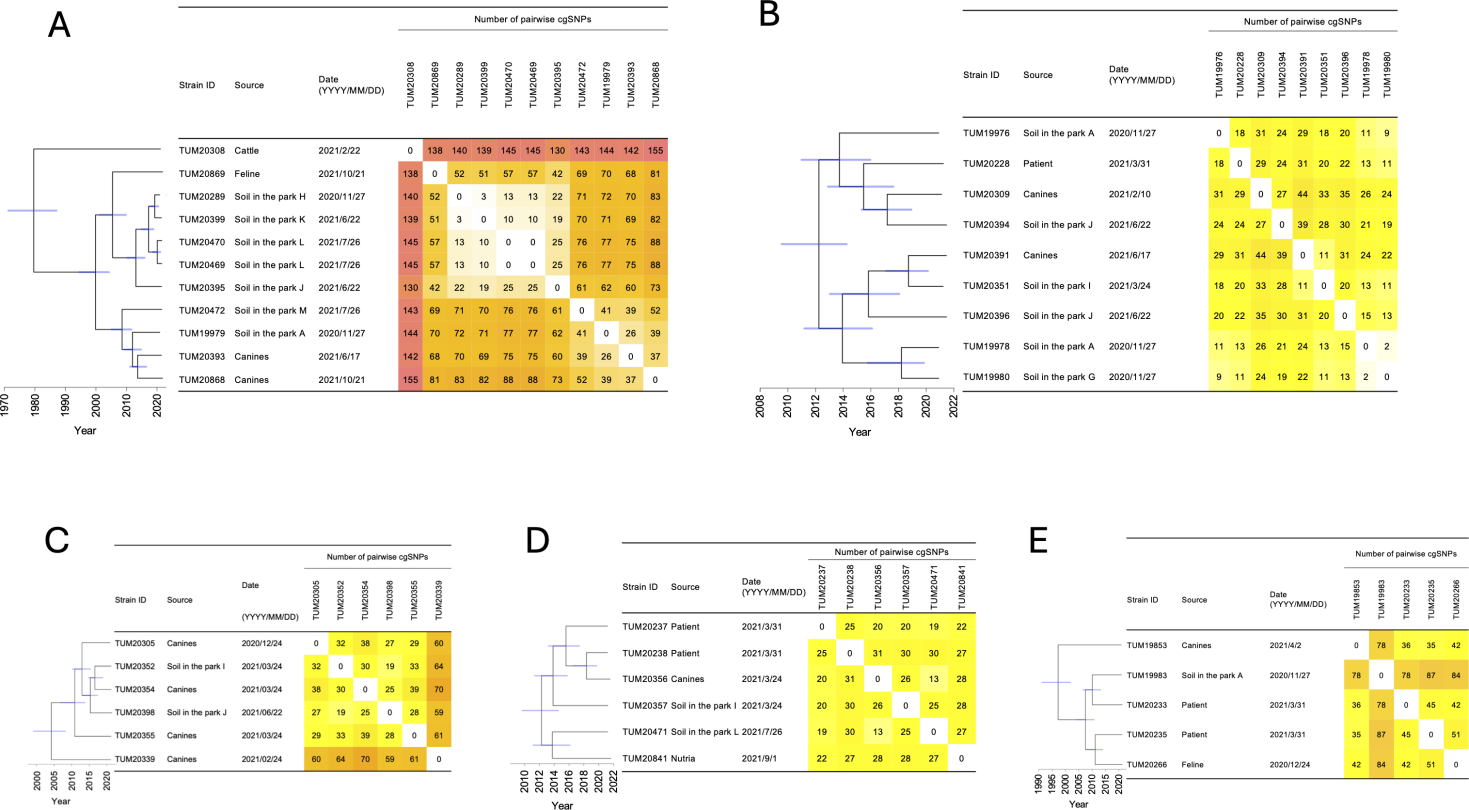
Dated phylogenetic analysis and genetic distances of *C. difficile* strains. Dated phylogenetic trees were inferred with BactDating from a maximum-likelihood phylogenetic analysis based on single-nucleotide polymorphisms (SNPs) in the core genome, after excluding regions inferred to result from homologous recombination. Horizontal bars at internal nodes indicate the 95% confidence intervals of estimated divergence times. Tree tips correspond to the strain isolation dates. (**A**) Phylogenetic tree of 11 strains of *C. difficile* ST2. A core genome region, amounting to 82.9% (3,453,358/4,167,076 bp), was shared with the genome of a reference strain, *C. difficile* 08ACD0030 (ST2). (**B**) Phylogenetic tree of 9 strains of *C. difficile* ST42. A core genome region, amounting to 96.8% (3,956,071/4,087,127 bp), was shared with the genome of a reference strain, *C. difficile* DH/NAP11/106/ST-42 (ST42). (**C**) Phylogenetic tree of 6 strains of *C. difficile* ST15. A core genome region, amounting to 94.2% (3,982,464/4,228,964 bp), was shared with the genome of a reference strain, *C. difficile* DSM 29,688 (ST15). (**D**) Phylogenetic tree of 6 strains of *C. difficile* ST100. A core genome region, amounting to 89.3% (3,502,452/3,922,278 bp), was shared with the genome of a reference strain, *C. difficile* FD009 (ST100). (**E**) Phylogenetic tree of 5 strains of *C. difficile* ST203. A core genome region, amounting to 87.6% (3,536,643/4,038,993 bp), was shared with the genome of a reference strain, *C. difficile* CBA7204 (ST203).

ST2, ST42, ST15, ST100, ST203, and ST28 were detected from multiple sources. Notably, ST42, ST100, and ST203 included isolates originating from canines, soil, and diarrheal outpatients. Core-genome SNPs analysis was performed to investigate the genetic relatedness among these isolates (Fig. 2A through E). To account for within-sample diversity, we established a provisional transmission-exclusion threshold of 13 SNPs. This value reflects (i) the 11 SNPs observed between the 2 ST42 isolates (TUM19976 and TUM19978) derived from the same soil sample (Fig. 1B) and (ii) the estimated evolutionary rate of 1.4 SNPs per genome per year expected over the 1-year study period (38). For ST2, the soil-derived isolate from Park H (TUM20289) and Park K (TUM20399) differed by only 3 SNPs, indicating close relatedness (Fig. 1A). Regarding ST42, the isolate from a diarrheal outpatient (TUM20228) differed by <13 SNPs from the soil isolate obtained from Park G (TUM19980) (Fig. 1B). In addition, soil isolates from Park A (TUM19976 and TUM19978) and Park G (TUM19980) also differed by fewer than 13 SNPs. These parks are geographically close and were frequently visited by the patient. Separately, a canine-derived isolate (TUM20391) and a soil isolate from Park I (TUM20351) exhibited a pairwise distance of 11 SNPs; the Park I was a regular walking area for the dog (Fig. 1B). These observations indicate close genomic relatedness among isolates from different sources within shared local environments, without implying epidemiological linkage or transmission. For ST100, the canine-derived isolate (TUM20356) and the soil-derived isolate from Park L (TUM20471) showed 13 SNPs, meeting the provisional threshold for potential relatedness (Fig. 1D). By contrast, ST15 and ST203 isolates showed no close genetic relationships, with all pairwise distances ≥30 SNPs (Fig. 1C and E).

## DISCUSSION

In this study, we examined the potential contribution of animals and environmental sources to CA-CDI in Japan. Our findings align with global observations indicating that CA-CDI can occur in individuals without traditional risk factors such as hospitalization or antibiotic exposure (21, 22). Although only 9.1% of CDI cases in Japan are classified as community-associated (20), the presence of genetically related strains in diarrheal outpatients, animals, and soil highlights the need to consider alternative transmission routes beyond healthcare settings. WGS revealed two notable genetic links among ST42 isolates: one between a patient-derived strain and soil from a park, and another between a canine-derived strain and soil from a different park. Limited contextual information obtained during routine clinical care and communication with the pet owner indicated park access by both the patient and the dog; however, no systematic exposure assessment or follow-up investigation was conducted.

Among the animal sample sources, the highest detection rate of toxigenic *C. difficile* was observed in companion animals, particularly canines. Previous studies have also reported a wide prevalence range in canines (4%–30%), with toxigenic strains frequently isolated (39–42). In livestock, our study demonstrated a relatively low detection rate, whereas the literature reports variable prevalence rates in calves (0.5%–94%), poultry (0.7%–57.4%), and swine (8.6%–100%), depending on the region (25, 26). The lower prevalence in livestock observed in this study may be partly attributed to the relatively older age of sampled animals, as younger animals tend to exhibit higher carriage rates (16, 25, 33, 35). Data on the prevalence of *C. difficile* in wild animals remain limited. RT057/010/005/029 has been detected in rodents on pig farms in the Netherlands, and RT078/002/014 has been detected in birds in Slovenia (43, 44). In this study, ST100 was isolated from a nutria, as well as patients, canines, and soil although they were not closely related. Notably, soil samples showed a particularly high detection rate (51.4%) with 68% of the strains harboring the toxin gene, consistent with previous reports identifying *C. difficile* in diverse natural environments (16, 36, 45–49).

ST2 was the most prevalent genetic lineage, detected across multiple sample types, particularly soil. Previous studies have also reported ST2, ST17, ST8, and ST37 as predominant in Japan (11, 20, 50). Additionally, ST42, ST100, ST203, and ST28 were detected in both patients and other specimens, while ST15 was detected in canines and soil (Table 2).

Analysis of antimicrobial resistance genes revealed the presence of fluoroquinolone resistance-associated mutations in approximately 40% of strains, including GyrA Thr82Ile and GyrB Ile139 variants. A previous study from Japan reported a moxifloxacin resistance rate of 100% among RT018/ST17 and RT369/ST81, compared with a rate of 14% for RT014/ST2 and 69% for RT002/ST8 (50). Although susceptibility testing was not conducted, the GyrA Thr82Ile mutation is a well-documented genetic marker of high-level fluoroquinolone resistance in *C. difficile*. In Japan, the overall use of fluoroquinolones, macrolides, and lincosamides is relatively low compared with many Western countries. According to reports from the Ministry of Health, Labour and Welfare, oral fluoroquinolones, macrolides, and lincosamides accounted for approximately 2.07, 3.45, and 0.02 defined daily doses per 1,000 inhabitants per day (DID), respectively, in 2023. Meanwhile, the use of injectable antibiotics remained minimal and stable over time (51). Despite this relatively modest antimicrobial usage, mutations associated with fluoroquinolone resistance were detected in isolates from animals and environmental sources. These findings suggest that antimicrobial resistance may present to some extent in *C. difficile* isolates derived from environmental and animal sources. No mutations associated with fidaxomicin resistance were detected in RpoB and RpoC.

To exclude genetically unrelated strains, we adopted a hypothetical cutoff of 13 SNPs based on the within-sample diversity observed among ST42 strains isolated from the same park soil (11 SNPs) and the previously reported estimated *C. difficile* evolutionary rate of 1.4 SNPs per genome per year (38, 52). In the One Health context, the ≤2 SNP threshold commonly used for direct human-to-human transmission in hospitals may be overly stringent (53); therefore, we selected a more relaxed cutoff suitable for our approximately 1-year study period. These SNP-based relationships should be interpreted as evidence of possible shared environmental reservoirs rather than direct transmission pathways.

Although previous studies have reported the presence of *C. difficile* in livestock, companion animals, and the environment, few have comprehensively examined the genetic relatedness of these sources to community-associated *C. difficile* using WGS and SNP-based phylogenetics. Our findings build on prior work, such as that of Eyre et al., which demonstrated that a substantial proportion of CDI cases had no direct epidemiological links, suggesting that environmental sources may play a larger role than previously recognized (53).

This study has some limitations. First, the geographic distribution of sampling sites was biased toward Tokyo and other regions accessible to collaborating institutions, which may limit the generalizability of the findings. Second, most livestock and companion animals sampled were relatively mature despite evidence that younger animals exhibit higher *C. difficile* carriage rates. Future studies should, therefore, include younger animals and a broader geographic range to improve detection and generalizability.

In addition, given the limited number of isolates and the exploratory nature of the study, the observed genomic relationships should be interpreted cautiously. Broader and longitudinal surveillance integrating human, animal, and environmental sampling will be required to more fully elucidate the ecological dynamics and potential reservoirs of *C. difficile* across human–animal–environment interfaces within a One Health framework.

In conclusion, our findings suggest that subsets of *C. difficile* ST42 circulate among humans, companion animals, and soil, highlighting the importance of a One Health approach in understanding the epidemiology of CA-CDI.

## MATERIALS AND METHODS

### Sample collection

Fecal and soil samples were collected in Japan between October 2020 and November 2021 for the isolation of *C. difficile*. Samples included feces from companion animals, livestock (cattle, poultry, and swine), wild animals, and diarrheal outpatients, as well as soil from public parks. Sampling locations were determined by the geographic areas in which collaborating institutions and field personnel were able to collect specimens under standardized conditions during the study period (Fig. 1). All samples were transported to the laboratory at 4°C and processed on the day of arrival or stored overnight at 4°C prior to processing.

#### Companion animals

Fecal samples from canines and felines were obtained from three veterinary clinics—two located in the Kanto region and one in the Kansai region—as well as from two household dogs kept at separate residences in Tokyo (one owned by an author and one owned by a patient’s family), yielding a total of five sampling sources (Fig. 1). At the veterinary clinics, samples were collected from animals presenting for routine health examinations, vaccinations, or minor non-gastrointestinal conditions. Fresh fecal samples were collected either by trained veterinary staff or by the owners using sterile spatulas and were immediately transferred into sterile screw-cap containers.

#### Livestock

Fecal samples from clinically healthy cattle, poultry, and swine were obtained from 17 cattle farms, 8 poultry facilities, and 4 swine farms across Japan (Fig. 1). All animals were raised for food production and were not companion animals. All samples were collected using sterile techniques, placed into sterile containers, and transported to our laboratory at 4°C. For cattle, fecal samples were collected either immediately before slaughter at slaughterhouses or from animals housed on production farms. Freshly voided feces were collected by farm staff under hygienic conditions. For swine, fecal samples were obtained immediately prior to slaughter at slaughterhouses using sterile techniques. For poultry, cecal contents were aseptically collected during processing at poultry production facilities. Individual cattle were identified using ear tags or farm identification numbers, whereas poultry flocks and swine were identified by farm and housing unit.

#### Wild animals

Rectal fecal samples were obtained directly from captured wild animals in the field using gloves and a sterile cotton swab with transport medium (SEEDSWABγ1 “Eiken,” Eiken Chemical Co., Ltd., Tokyo, Japan). Animals were sampled immediately after capture during harmful wildlife control activities, hunting, or academic capture procedures, all conducted with approval from the prefectural government. No fecal samples were collected from the ground, and animals were not transported to veterinary clinics for sample collection. Samples were collected at multiple locations across Gifu Prefecture—including forest edges, agricultural fields, riverbeds, and peri-urban green spaces—as well as at a single village site in Nagano Prefecture.

#### Park soil and sandbox soil

Soil samples were collected from eight geographically distinct public parks in Tokyo. Parks were selected based on geographic distribution and the presence of either children’s sandboxes or accessible open soil areas. When available, two types of soil were collected; sandbox soil: the top 2–3 cm of sand from sandbox structures, and non-sandbox topsoil: the top 2–3 cm of exposed soil within park grounds. Approximately 10 g of soil from a 10 cm² area was collected using sterile scoops and placed in sterile screw-cap containers.

#### Outpatients

Outpatients presenting with diarrhea and undergoing routine stool culture testing, during which *C. difficile* was isolated at two medical facilities in Tokyo, were consecutively included in the study. Stool samples were obtained as part of the patients’ routine diagnostic evaluation, with no additional sampling performed for research purposes. CDI was defined as the presence of diarrheal symptoms with a consistency of Bristol Stool Scale type ≥5, a positive stool test result for toxins, or isolation of toxigenic strains from stool (11). CA-CDI was defined as symptom onset in the community occurring >12 weeks after last discharge from a healthcare facility (11).

### Isolation and identification of *C. difficile*

The isolation of *C. difficile* was performed using previously described methods (54, 55), with minor modifications. Regarding cattle, poultry, swine, felines, canines, and wild animals, fecal samples were treated with alcohol to select for spores as described (32, 34, 56). *C. difficile* was then isolated on cycloserine-cefoxitin mannitol agar-Ex (CCMA-EX, Nissui Pharmaceutical, Tokyo, Japan). For the isolation of *C. difficile* from soil, 5 g of the samples was mixed with 5 mL of 99.5% ethanol and allowed to stand at room temperature for 30 min. The contents were then mixed, and the supernatant was collected after the sand had settled. Subsequently, the supernatant was centrifuged at 1,600 × *g* for 30 min at room temperature. A 200 μL aliquot of the resulting pellet was inoculated onto CCMA-EX agar and into 2 mL of Brain Heart Infusion medium supplemented with L-cysteine (BHIS medium; composed of 52 g BHI [BD, Franklin Lakes, NJ, USA], 5 g yeast extract [Gibco, Thermo Fisher Scientific, Waltham, MA, USA], and 10 mL of 10% L-cysteine [Wako Pure Chemical Industries, Osaka, Japan] in 1 L of distilled water) and then incubated anaerobically at 35°C for 48 h (36). Based on the characteristic colony morphology (yellow, ground glass appearance) and odor (horse dung smell), isolated colonies were purified by re-streaking. When colonies with distinct morphologies were observed within a single specimen, each morphologically distinct colony type was separately subcultured and analyzed to assess the presence of multiple sequence types within the same sample. Species were then identified as *C. difficile* by matrix-assisted laser desorption/ionization time-of-flight mass spectrometry (MALDI-TOF MS) using the MALDI Biotyper (Bruker Daltonics GmbH, Bremen, Germany) with a score value of 2.000 or more. Isolates revealing high-confidence identification were stored at −80°C until further molecular analysis.

### Draft whole-genome sequencing and data analysis

Bacterial DNA was extracted using achromopeptidase and phenol-chloroform treatment (57) and purified using a FastGene Gel/PCR Extraction Kit (Nippon Genetics Co., Ltd) to perform the draft WGS of *C. difficile* isolates. DNA libraries were prepared using the QIAseq FX DNA Library Kit (QIAGEN, Hilden, Germany) and sequenced on the MiSeq platform (Illumina, Inc., San Diego, CA, USA) with 300-bp paired-end reads. Raw reads were processed using Trimmomatic version 0.39, trimming adapter sequences and low-quality bases not passing a quality score of Q20 or higher. The following parameters were applied: LEADING:20, TRAILING:20, SLIDINGWINDOW:4:15, MINLEN:50, and HEADCROP:5 (58, 59). Trimmed reads were assembled using the SPAdes version 3.15.2 (58, 59) with paired-end reads. Species identification was performed using KmerFinder (version 3.2) (https://cge.food.dtu.dk/services/KmerFinder/). Multilocus sequence typing (MLST) was performed using the *C. difficile* PubMLST scheme (https://pubmlst.org/). Toxin genes (*tcdA*, *tcdB*, *cdtA*, *cdtB*, and *tcdC*) and antimicrobial resistance-associated genes (*gyrA*, *gyrB*, *rpoB*, and *rpoC*) were detected using the Basic Local Alignment Search Tool (BLAST; https://blast.ncbi.nlm.nih.gov/Blast.cgi) (37). Genetic variations in the toxin genes were identified by comparison with reference genomes of *C. difficile* strain 630 (*tcdA*^+^, *tcdB*^+^, *cdtA^−^*, and *cdtB^−^*; GenBank accession no. NC_009089) and strain CD196 (*tcdA*^+^, *tcdB*^+^, *cdtA*^+^, and *cdtB*^+^; GenBank accession no. NC_013315) (60). Regarding antimicrobial resistance, amino acid substitutions previously associated with reduced susceptibility to fluoroquinolones (GyrA T82; GyrB I139, S366, D426) and fidaxomicin (RpoB V1143, Q1149; RpoC R89, R326) were analyzed (57). Additionally, MLSB resistance genes, *erm*(B) and *erm*(T), were identified using ResFinder (version 4.1) (https://genepi.food.dtu.dk/resfinder).

### Core-genome SNP-based phylogenetic analysis

Core-genome SNP-based phylogenetic analysis was conducted using MiSeq read data. The reads were aligned to the reference strains’ genome sequences using the Burrows-Wheeler Aligner with the “SW” algorithm (61). The reference genomes were selected for each ST of *C. difficile* in this study, with *C. difficile* 08ACD0030 (GenBank accession number CP010888) for ST2, *C. difficile* DSM 29,688 (GenBank accession number CP019858) for ST15, *C. difficile* DH/NAP11/106/ST-42 (GenBank accession number CP022524) for ST42, *C. difficile* FD009 (GenBank accession number BIND01000001) for ST100, and *C. difficile* CBA7204 (GenBank accession number CP029566) for ST203. Core-genome alignments were extracted using SAMtools (version 1.9) mpileup with base alignment quality adjustment disabled and reference genome specified explicitly (62). SNPs and consensus sequences were identified with VarScan (version 2.3.9) using the mpileup2cns function with default parameters (63). A temporal phylogenetic tree was constructed based on the maximum likelihood method using PhyML (version 3.3.20,241,207) with the general time reversible (GTR) substitution model and 100 bootstrap replicates (64). The tree was used as the initial input for ClonalFrameML (version 1.12) to homologous recombination events that introduced DNA fragments from outside the phylogenetic cluster, thereby generating a recombination-corrected clonal phylogeny (65). A phylogenetic tree based on core-genome SNPs, excluding homologous recombination regions inferred by ClonalFrameML, was reconstructed using the maximum likelihood method implemented in RAxML (version 8.2.12) with the GTR substitution model and 1,000 bootstrap replicates (66). All trees were visualized using Figtree (version 1.4.4; https://github.com/rambaut/figtree/releases/tag/v1.4.4). The number of SNPs in the core genome was calculated using Snp-dist (version 0.7.0; https://github.com/tseemann/snp-dists). Dated phylogenetic trees were inferenced using BactDating under the “arc” (autocorrelated relaxed clock) model, assuming a nucleotide substitution rate of 5× 10^−7^ substitutions per genome per year (67, 68).

## Data Availability

Draft genome sequences have been deposited in the NCBI database under BioProject accession number PRJNA1302146.

## SUPPLEMENTAL MATERIAL

Table S1 aem.00397-26-s0001.xlsx Toxin-encoding gene and antibiotic resistance gene profiles of the isolated *Clostridioides difficile* strains.

ASM does not own the copyrights to Supplemental Material that may be linked to, or accessed through, an article. The authors have granted ASM a non-exclusive, world-wide license to publish the Supplemental Material files. Please contact the corresponding author directly for reuse.
