# Correction for Yoshizawa et al., “One Health genomic insights into environmental and animal reservoirs of community-associated *Clostridioides difficile* in Japan”

**DOI:** 10.1128/aem.01302-26

**Published:** 2026-07-28

**Authors:** Sadako Yoshizawa, Kohji Komori, Kotaro Aoki, Tomoka Sawa, Yoshimasa Sasaki, Yasuhisa Kurosaki, Yoshiteru Murata, Tomoko Kasai, Nobuaki Mori, Tetsuo Asai, Yoshikazu Ishii, Kazuhiro Tateda

## AUTHOR CORRECTION

Volume 92, no. 6, e00397-26, 2026, https://doi.org/10.1128/aem.00397-26. Following a reader inquiry regarding the toxin profile of ST37, we re-examined the whole-genome sequencing data and identified errors in the interpretation of *tcdA* detection results affecting ST37 and ST81.

Results, 4th paragraph: “ST2, ST42, ST11, ST37, ST47, ST58, ST81, ST185, and ST224” should read “ST2, ST42, ST11, ST47, ST58, ST185, and ST224.”

Results, 4th paragraph: “ST 919 was positive” should read “ST37, ST81, and ST919 were positive.”

Table 2, toxin gene profile for ST37: “*tcdA*^+^ *tcdB*^+^ *cdtA*^−^ *cdtB*^−^” should read “*tcdA*^−^ *tcdB*^+^ *cdtA*^−^ *cdtB*^−^.”

Table 2, toxin gene profile for ST81: “*tcdA*^+^ *tcdB*^+^ *cdtA*^−^ *cdtB*^−^” should read “*tcdA*^−^ *tcdB*^+^ *cdtA*^−^ *cdtB*^−^.”

Table S1, *tcdA* column for isolate no. 29, ST37: “+” should read “−.”

Table S1, *tcdA* column for isolate no. 41, ST81: “+” should read “−.”

These corrections do not affect the phylogenetic analyses, SNP analyses, results, discussion, or conclusions of the study.

